# Need for HTA supported risk factor screening for hypertension and diabetes in Nepal: A systematic scoping review

**DOI:** 10.3389/fcvm.2022.898225

**Published:** 2022-08-01

**Authors:** Chiranjivi Adhikari, Rojana Dhakal, Lal Mani Adhikari, Bijaya Parajuli, Khem Raj Subedi, Yeshoda Aryal, Arjun Kumar Thapa, Komal Shah

**Affiliations:** ^1^Department of Public Health, SHAS, Pokhara University, Pokhara, Nepal; ^2^Indian Institute of Public Health Gandhinagar, Gandhinagar, India; ^3^Department of Nursing, School of Health and Allied Sciences, Pokhara University, Pokhara, Nepal; ^4^Department of Life and Health Sciences, University of Nicosia, Nicosia, Cyprus; ^5^Health Research and Social Development Forum International, Kathmandu, Nepal; ^6^Ministry of Health and Population, Gandaki Province, Myagdi Health Office, Myagdi, Nepal; ^7^Department of Economics, Far Western University, Tikapur Multiple Campus, Kailali, Nepal; ^8^Ministry of Health and Population, Kathmandu, Nepal; ^9^Department of Economics, School of Humanities and Social Sciences, Pokhara University, Pokhara, Nepal

**Keywords:** health technology assessment (HTA), cardiovascular, risk factor, hypertension, diabetes, screening, review, Nepal

## Abstract

**Objective:**

Health Technology Assessment (HTA) is a comprehensive and important tool for assessment and decision-making in public health and healthcare practice. It is recommended by the WHO and has been applied in practice in many countries, mostly the developed ones. HTA might be an important tool to achieve universal health coverage (UHC), especially beneficial to low-and-middle-income countries (LMIC). Even though the Package for Essential Non-communicable Diseases (PEN) has already been initiated, there is a clear policy gap in the HTA of any health device, service, or procedure, including the assessment of cardiovascular risk factors (CVRFs) in Nepal. Hence, we carried out the review to document the HTA supported evidence of hypertension and diabetes screening, as CVRFs in Nepal.

**Materials and methods:**

We searched in PubMed, Cochrane, and Google Scholar, along with some gray literature published in the last 6 years (2016–2021) in a systematic way with a controlled vocabulary using a well-designed and pilot tested search strategy, screened them, and a total of 53 articles and reports that matched the screening criteria were included for the review. We then, extracted the data in a pre-designed MS-Excel format, first in one, and then, from it, in two, with more specific data.

**Results:**

Of 53 included studies, we reported the prevalence and/or proportion of hypertension and diabetes with various denominators. Furthermore, HTA-related findings such as cost, validity, alternative tool or technology, awareness, and intervention effectiveness have been documented and discussed further, however, not summarized due to their sparingness.

**Conclusion:**

Overall, the prevalence of DM (4.4–18.8%) and HTN (17.2–70.0%) was reported in most studies, with a few, covering other aspects of HTA of DM/HTN. A national policy for establishing an HTA agency and some immediately implementable actions are highly recommended.

## Introduction

Health technology assessment (HTA) is a multidisciplinary approach incepted during the 1970s for assessing technologies such as drugs, devices, procedures, settings of care, services or programs, including the screening in health care with the collection of data from epidemiological, clinical efficacy, quality of life, service utilization, and cost (both health systems' and patients' out of pocket payments) studies.

HTA assists in evidence informed health policy making on the introduction and use of health technologies. Since any state or country has competing priorities, policy-makers often decide what is the best buy for the available evidence-base and the budget, which can further help as suggested by the WHO, in developing the health benefit package (HBP) for any state or country (1). At least two dimensions (if not three) of universal health coverage?the proportion of the cost and the services covered are directly linked to and implicated by HTA. Countries like the USA, Australia and Canada have been benefiting from HTA for decades, whereas Asian countries like South Korea, Japan, China, and India (recently) are gaining momentum (2). As per a survey carried out by WHO in 2015, Nepal has neither rendered a national HTA body nor is there clarity on how HTA is used in decision-making (3).

According to the global burden of disease study (GBOD) conducted in Nepal in 2017, non-communicable diseases were responsible for two-thirds of all deaths (66%), while high blood pressure and high fasting blood glucose levels were responsible for 14 and 10% of all deaths, respectively (4). Screening can avert disability adjusted life years (DALYs) of 2.33–3.1 in the case of hyperglycaemia (5) and is cost-effective in both hyperglycaemia and hypertension (5, 6).

Diabetes is a metabolic disorder identified by screening using technologies like laboratory tests that include fasting plasma glucose, 2-h (2-h) post-load plasma glucose after a 75-g oral glucose tolerance test (OGTT); hemoglobin A1c (HbA1c); and random blood glucose in the presence of signs and symptoms. Diabetes is defined as fasting plasma glucose of 7.0 mmol/L (126 mg/dl), 2-h post-load plasma glucose of 11.1 mmol/L (200 mg/dl) (5), HbA1c ≥ 6.5% (48 mmol/ml); or a random blood glucose of ≥11.1 mmol/L (200 mg/ dl) in the presence of signs and symptoms. If elevated values are detected in asymptomatic people, repeat testing, preferably with the same test, is recommended as soon as practicable on a subsequent day to confirm the diagnosis (7).

Raised blood pressure can be identified at either a clinic, home-based, or ambulatory screening, and the diagnosis can be made with repeated measurements at 1–4 week intervals. In addition to this, blood tests, echocardiography, urine dipstick, albumin, and liver functions tests are other additional tests that are carried out in hypertensive suspects or diagnosed cases (8). As a part of assessing cardiovascular risk, mean systolic blood pressure should be measured over two separate occasions. For those who are already on anti-hypertensive medicine, the most recent recorded pretreatment value should be adopted as given by the National Institute for Health and Care Excellence/British Hypertension Society (NICE/BHS) guideline (9).

The population-based screening for diabetes and hypertension at a frequency ranging from every year to every 20 years was not cost-effective at the present level healthcare systems in India. However, it was estimated that screening at 3-to-5-year intervals could be cost-effective if the proportion of newly diagnosed and treated patients increased by more than 20%. Providing population-based screening of those two disease conditions through primary health centers (PHC) could be cost-effective; therefore they need to be strengthened at PHC level (10). A comparative study of BP measured in Shardaben General Hospital, India by digital and aneroid sphygmomanometers showed mean SBP as 108.92 ± 15.14 and 109.66 ± 16.81, and mean DBP as 76.20 ± 12.25 and 78.02 ± 14.35 mm of Hg, respectively, justifying that the bias for mean SBP and DBP was clinically non-significant and both instruments can be used interchangeably (11).

Diagnosing hypertension by sphygmomanometer is considered the gold standard as it has a high level of accuracy. A study from West Bengal showed that the aneroid device had better accuracy than the digital device as compared to the mercury sphygmomanometer (12). The mercury sphygmomanometer, which was widely used in the past to measure blood pressure in an office or clinic, has now been largely phased out in US hospitals, leading to the use of non-mercury, aneroid, or hybrid manometers in clinic and hospital settings (13, 14). Ambulatory monitoring was the most cost-effective strategy for all ages of men and women after an initial raised reading at the clinic or at home for blood pressure diagnosis, and so, it should be taken as a reference standard (15, 16).

Similarly, other instruments like 12-lead portable ECG devices for the screening of cardiovascular diseases in Ahemdabad found that screening at PHC by ECG saves 2.9 lives per year at an incremental cost of 89.97 USD, yielding a cost-effectiveness ratio of 31.07 USD, so the facility to screen cardiac abnormality at PHC level is highly recommended for risk adults and symptomatic cases (17). It coincides with the US preventive services task force for screening for prediabetes and type 2 diabetes at ages 35–70 years in a primary setting, which has a moderate net benefit. It was suggested that screening every 3 years may be a reasonable approach for adults with normal blood glucose as the initial normal glucose test result is limited. The diagnosis of type 2 diabetes should be confirmed with repeated tests. Now, the US Preventive Services Task Force (USPTSF) changed their practice of 2015 recommendation and lowered the initial age of screening from age 40 to 35 among adults who were obese or overweight (18).

In Bhutan, an economic evaluation of the World Health Organization (WHO)'s Package of Essential Non-communicable diseases (PEN) found that the ambulatory but high-risk screening (where people who are overweight, obese, or >40 years for DM and/or HTN, visiting primary care facilities) represents good value for money compared to “no screening” (19). However, if performed on a regular basis and taking into account the specific population group and the existing non-disease to disease conversion rate, opportunistic high-risk approach screening may also yield high results (but may not confirm cases) (20).

The Indian diabetes risk score (IDRS) was used in a study from Tamil Nadu, India to determine the prevalence of type 2 diabetes high-risk cases. Use of the IDRS could reduce the cost of diabetes screening in India and so, being suggested to use in mass screening (21). Insulin pumps or glucose sensors appear cost-effective, particularly in populations with higher HbA1c levels and rates of hypoglycaemia. However, the cost-effectiveness for combined insulin pumps and glucose sensors was less clear (22).

A study from Kuwait assessing type 2 diabetes and hypertension using machine-learning among 89,858 diabetics, 58,745 hypertensives, and 30,522 co-morbids shows that >85 and >90% accuracies were achieved for diabetes and hypertension, respectively using simple non-laboratory-based parameters. Talking more about the prediction, ethnicity is seen as a significant factor to the predictive models such as Asian-specific models and assessments perform even better (23). Population based screening for high- risk strategies can prevent cardiovascular disease. Also, it was suggested that where resources are limited, further advice be taken to take a total risk approach to identify several risk factors targeting to high-risk group to identify hypertension. Targeting high-risk groups for screening can help reduce costs because resources are not spent on the entire population. Early detection results in timely treatment and management of risk factors, which ultimately assists in reducing morbidity and mortality and reducing health-related costs (24).

A 540-person study in rural West Virginia was designed to identify diabetic risk using an HbA1c test and a diabetes risk assessment tool. Results showed that 61.8% of participants were at high risk for diabetes. It shows that community-based screenings are an effective way to assess diabetes risk (25).

Because cardiovascular risk factors were prevalent among Nepal's rural population, comprehensive intervention targeting all risk factors should be planned and implemented to reduce the burden of CVD in Nepal (26). Nepal has yet to work to implement health policies to tackle CVD or other NCDs. Recently, CVD management has been focused on treatment as there has been a rise in the availability of interventional cardiology and cardiothoracic surgery services (27). An assessment of health facilities for the implementation of a non-communicable disease package in Nepal among 92 health facilities in Kailali and Ilam districts revealed the gaps in the capacity of health institutions and the system in terms of training, supply, equipment, and diagnostics (28).

According to WHO, HTA should include the complete range of interventions or technology, and not be limited to just one. Technologies and methods considered in HTA include safety, clinical effectiveness, equity issues, ethical issues, feasibility, and acceptability of the health care system by providers and patients (29). Based on serum HbA1c levels, diabetes screening was offered at the Durham Veterans Affairs Medical Center for people aged 45–64 years old. Participants with an HbA1c ≥ 6.0% were invited for a follow-up measurement of blood pressure fasting plasma glucose, and health-related quality of life (HRQoL) were measured. Those with HbA1c ≥ 7.0% or fasting plasma glucose ≥7 mmol/dl are diagnosed as having unrecognized diabetes. There was no difference in the HRQoL of patients diagnosed with diabetes and those not diagnosed with diabetes. Screening for diabetes has minimal, if any, “labeling” effect with respect to quality of life (30).

Hypertensive disorders are the risk factors for cardiovascular diseases in Nepal, and there is a wide gap and socio-economic disparity in hypertension management. In Nepal, the overall prevalence of high blood pressure was 19.6 percent. Furthermore, less than one-third of the population was treated, and <20% had their blood pressure under control. Wealth and education-based inequalities in awareness, treatment, and control measures of raised BP were significantly high in urban and rural areas (31). In this scenario, Nepal has developed PEN protocols and initiated the program in 2016 (32). These signify (but are not limited to) the systematic assessment of equity, cost, clinical efficacy, safety, ethically sound, feasible, and acceptability of health devices, procedures, and services before their population-wide applications.

## Materials and methods

### Review framework

We used the following methodological framework to conduct this scoping review; (1) Identifying review questions, (2) Creating review objectives, (3) Establishing eligibility criteria, (4) Creating a search strategy and identifying search sources, (5) Screening records and data extraction, and (6) Evidence synthesis.

### Search strategy

A comprehensive search was conducted using PubMed, Google Scholar, and the Cochrane databases, with publication date limits of the last 6 years (2016–2021). We also searched for reports, theses, and abstracts in gray literature and in citation searches.

A search strategy was finalized after developing a search strategy in consultation with the experts in the field. We searched with keywords such as screening, hypertension, diabetes, hyperglycaemia, Nepal, non-communicable, review, raised blood pressure, surveillance in the title, abstract, and keywords (ti; ab; kw); and with the medical subject heading (MeSH) descriptors such as heart disease risk factor, diagnostic techniques, blood pressure, diabetes mellitus. We searched on the basis of outcome indicators as illustrated in Table 1.

**Table 1:** PICO indicators and criteria for scoping review.

**PICO/Indicator**	**Criteria**
Study design	Published qualitative and quantitative data related to blood pressure and blood sugar assessment from study designs-case series, cross-sectional studies, cohort studies, RCTs, pilot trials, screening costing and economic evaluation, health technology assessment of diabetes and hypertension screening
Population	Clinical, co-morbid or healthy population aged 18 years or above who have undergone any type of screening or assessed in survey or surveillance in Nepal
Intervention	Screening, surveillance or survey
Comparator	Having control or standard treatment or placebo or no comparator
Outcome	Any of the followings (Qualitative and/or Quantitative finding) 1. From screening: any of true positive rate (TPR), true negative rate (TNR), false positive rate (FPR), false negative rate (FNR), sensitivity, specificity, yield, positive predictive value (PPV), negative predictive value (NPV). 2. Ratio of screened +ve to screened -ve (among the total visited to surveillance center) 3. From Screening, Survey or Surveillance: Number of subjects needed to screen or survey for one case of DM or HTN 4. Costing per case identifying 5. Costing per individual screening 6. Incremental cost-effectiveness ratio (ICER) 7. Proportion of hypertension (95% CI) 8. Proportion of diabetes mellitus (95% CI) 9. Proportion of hypertension awareness among the hypertensives 10. Proportion of diabetes awareness among the diabetics 11. Proportion of taking medication among the hypertensives 12. Proportion taking medicines among the diabetics 13. Proportion of controlled BP among the hypertensives 14. Proportion of controlled sugar among the diabetics 15. Health technology assessment (HTA) 16. Ethics in screening of DM and HTN 17. Quality of life (QoL) of screening of DM and HTN 18. Willingness to pay (WTP) and cost of screening of DM and HTN 19. Capacity to pay (CTP) of screening of DM and HTN
Published duration	Last 6 years (2016–2021)

### Study selection

**Figure 1:**
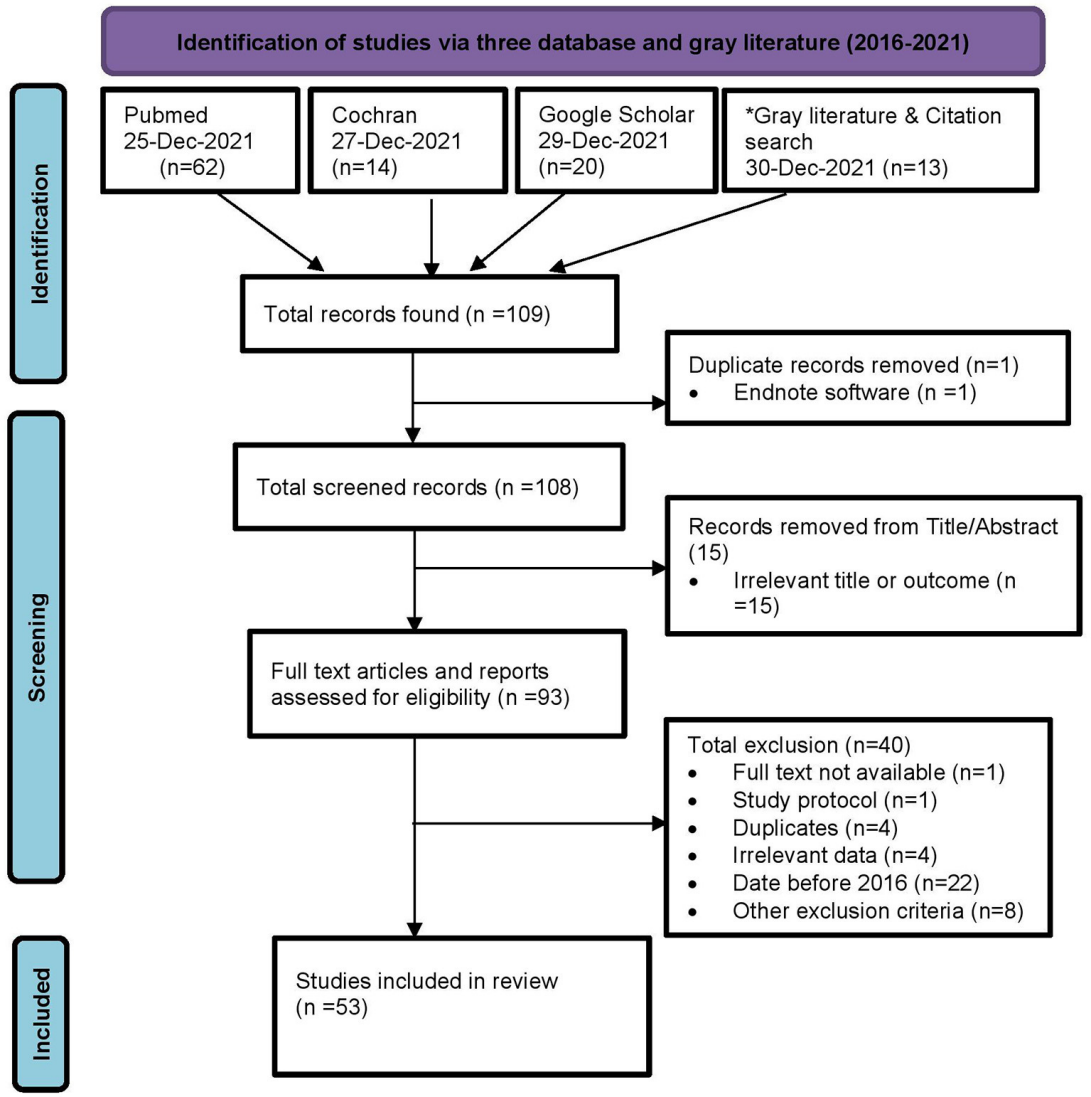
PRISMA chart of screening and included studies. *Gray literature search includes Shahid Gangalal National Heart Center (2), Mrigendra Samjhana Medical Trust (MSMT) (1), National Health Research Council (2), Ministry of Health and Population, Dept. of Health Services (3), Others (3), and Citation search (2).

After the strategy was finalized among all, a researcher, having trained and previous experience in systematic search and evidence synthesis, independently screened studies for eligibility and relevance. Studies were considered to be eligible if they had any of the outcome indicators as illustrated in Figure 1, including abstracts and reports. However, we excluded the protocols.

### Data extraction and data synthesis

Three different data extraction grids were developed by two review authors (CA and KS) and then sent to two authors (LMA and BP) for data extraction, from whom CA again obtained and compiled the data. Information regarding authors, country, study year, study type, sample size, geography, sex, age, ethnicity, and outcomes measures (Table 2), including key findings, and strengths and limitations (Table 3), was extracted from 53 individual studies (Figure 1), which were included in the review.

**Table 2:** Characteristics of the included studies.

**SN**	**References**	**Study type (Screening/survey/** **surveillance) and/or cross-sectional/longitudinal**	**Study detail (sample)**	**Study detail (geography/** **districts)**	**Population characteristics (Sex, age)**	**Population characteristics (caste/ethnicity)**	**Population characteristics (healthy, clinical)**	**Outcome measured**	**Remarks (publication type)**
1	Acharya et al. (33)	Opportunistic screening campaign	11486	Nepal	18 years and above, female- 6,568 (57%)	Not available (NA)	Healthy and clinical (on medications), both	• Proportion of hypertension; • Proportion of hypertension awareness among the hypertensives; • Proportion of persons on medication among the hypertensive	Conference abstract book
2	Adhikari (34)	Screening of cardiac patients using the invasive and non-invasive technology, Cardiac Camps (through May measurement month screening)	Varying (depends upon the screening technology)—For May measurement month screening Camps-1857	Nepal (facility-based)	All ages	Not available (NA)	Clinical and healthy	• Proportion of hypertension-camp based	Annual report
3	Agho et al. (35)	Secondary analysis of NDHS 2016	14,857 (males: 6,245 and females: 8,612)	Nepal	15 years and above	Not available (NA)	Clinical and healthy	• Prevalence of prehypertension and hypertension	Original article
4	Bhattarai et al. (36)	Secondary analysis	NA	Nepal	General population	NA	Clinical and healthy	• Proportion of contribution of CVD in mortality and DALYs	Original article
5	Bist et al. (37)	Survey	5593	Nepal	15–69 yrs.	NA	Clinical and healthy	• Proportion of raised blood pressure • Proportion of raised blood glucose	Original article
6	Brewis et al. (38)	Secondary analysis of NDHS 2016	14842	Nepal	General population	NA	Clinical and healthy	• Proportion of raised blood pressure	Research article
7	Aryal et al. (39)	Survey	521	Mustang and Humla (Mountain)	30 years and above	Tibetans and Khas-Aryas.	Clinical and healthy	• Proportion of raised blood pressure • Proportion of prediabetic and diabetic	Research article
8	Das Gupta et al. (40)	Secondary analysis of NDHS 2016	13,393	Nepal	18 years and above	General population	Clinical and healthy	• Proportion of hypertension awareness among the hypertensive • Proportion of using antihypertensive use among the hypertensive • Proportion of controlled BP among the hypertensive	Research article
9	Das Gupta et al. (41)	Secondary analysis of NDHS 2016	13,393	Nepal	18 years and above	General population	Clinical and healthy	• Prevalence of prehypertension and hypertension	Research article
10	Datta and Humagain (42)	Secondary analysis of NDHS 2016	3,778	Nepal	15–49 years married women	General population	Clinical and healthy	• Prevalence of prehypertension and hypertension	Research article
11	Dhungana et al. (43)	Secondary analysis of NCD Survey 2018	8,931	Nepal	20 years and above	General population	Clinical and healthy	• Prevalence of comorbidity with hypertension and diabetics	Research article
12	Dhungana et al. (44)	Survey	347	Sitapaila VDC, Kathmandu	18–70 years excluding self- reported CVD and pregnant women	General population	Clinical and healthy	• Prevalence of hypertension • Prevalence of diabetes	Research article
13	Ene-Iordache et al. (45)	Secondary analysis of 12 countries (LMICs)	Total = 75,058; Nepal = 21066	12 countries	18 years or older	General population	Clinical and healthy	• Prevalence of hypertension • Prevalence of having awareness of disease onset (HTN and Diabetes)	Research article
14	Ghimire et al. (46)	Secondary analysis of STEPS 2013	526	Nepal	60–69 years	General population	Clinical and healthy	• Prevalence of hypertension • Prevalence of diabetes	Research article
15	Ghimire et al. (47)	Secondary analysis of STEP Survey 2013	4,200	Nepal	45–69 years	NA	General population	• Prevalence of raised blood pressure	Research article
16	Gyawali et al. (48)	Review article	34 studies	Nepal	NA	NA	NA	• Costing per case treatment • Prevalence of DM type 2	Research article
17	Paudel et al. (49)	Descriptive cross-sectional study	977 family members of 290 households	Kaski district, Nepal.	• Male 46.4%, Female 53.6% • Adult 79.5%, Elderly 20.5%	Brahmin 41.4%, Chhetri 17.4%, Dalit 88%, Gurung 18.2%, Others 14.2 %	Healthy and clinical (on medications), both	• Proportion of hypertension • Proportion of hypertensive patients under medication • Proportion. of hypertensive patients who had their blood pressure under control after taking the medications	Research article
18	Peoples et al. (50)	Mixed-method Survey	114 Quantitative; 20 Qualitative	Ten PHC facilities across two regions of Nepal: five in Kailali district and five in Sindhuli district.	• Male (51%) female (49%) of over 18 years of age	NA	Have ever been diagnosed with at least one of the following conditions: heart disease, stroke, hypertension, and/or diabetes	• Assessment of the use and perception of PHC services in Nepal among people living with Cardio metabolic diseases for primary and secondary prevention of cardiovascular disease	Research article
19	Rana et al. (51)	Secondary analysis of NDHS 2016	13,436	Nepal	Male = 5,645, Female = 7,790, Population of age 18 years and above	NA	Healthy	• Prevalence of hypertension • Factors associated with hypertension	Published article
20	Rai et al. (52)	Cross-sectional	1,905	Kathmandu, Nepal	Male = 60.3%, Female = 39.7%	NA	Clinical	• Proportion of hypertension • Proportion of diabetes • Proportion of combined diabetes and hypertension	Research article
21	Rauniyar et al. (31)	Secondary analysis of NDHS 2016	802,167 (787,713 in India, 14,454 in Nepal)	India and Nepal	Nepal • (Male = 58.2%, Female = 41.8%) • Age between 15 and 49 years	NA	Healthy and clinical (on medications), both	• Prevalence, awareness, treatment, and control of hypertension • Wealth-based inequality in prevalence and management of hypertension	Research article
22	Sainju et al. (53)	Cross sectional	1,243	Sindupalchowk district	Female 70%, Male 30%, 18 and above age	NA	Healthy	• Prevalence of pre hypertension and hypertension.	Research article
23	Saito et al. (54)	Cross-sectional	9,177 individuals residing in 1,997 households	Kathmandu, Nepal	• Female 49.1%, Male 51.0% • All ages	NA	Healthy	• Prevalence of non-hypertension and diabetes	Research article
24	Paudel et al. (55)	Secondary analysis of STEPS 2013	1,977	Nepal	15–69 years	NA	Healthy and clinical (on medications), both	• Prevalence of type 2 diabetes mellitus • Factors associated withType-2 Diabetes Mellitus	Journal Pre-proof document
25	Gyawali et al. (56)	Population-based cross-sectional survey	2,310	Lekhnath Municipality of Nepal	(Female 68%, Male 32%) 25 years or above	Upper caste = 54%, Janajati = 32%, Others = 14%	Healthy	• Prevalence of type 2 diabetes • Factors associated with Type 2 diabetes Awareness, treatment, and control status of type 2 diabetes	Research article
26	Shrestha et al. (57)	Systematic review	15 studies were included in the qualitative and quantitative analysis	Nepal		NA	Having prevalence of T2DM and/or details such as risk factors	• Prevalence of T2DM, pre-diabetes, and impaired glucose tolerance • Factors associated with T2DM	Research article
27	Shrestha et al. (58)	Review	14 eligible studies that comprised a total of 44,129 participants and 3,517 diabetes cases	Nepal	≥20 years old	NA	Healthy, clinical (on medication) l	• Prevalence of Prediabetes and diabetes awareness, treatment and control of diabetes	Research article
28	Shrestha et al. (59)	Hospital based cross sectional	2,256	Bhaktapur district, Nepal	Age: Between 40 and 69 years old.	NA	Outpatients	• Prevalence of hypertension and pre-pre hypertension • Factors associated with hypertension and pre-pre hypertension	Research article
29	Silvanus et al. (60)	Community based, cross-sectional, analytical study	256	Budhanilkantha Municipality, Kathmandu district	170 female, 86 male Age: 50 years old and above	NA	Healthy	• Prevalence of diabetes • Classification of risk for developing diabetes	Research article
30	Silvanus et al. (61)	Community based, cross-sectional, analytical study	162	Budhanilkantha municipality in Kathmandu District		NA	Healthy	• Prevalence of undiagnosed diabetes and prediabetes	Research article
31	Tan et al. (62)	Qualitative study	23 IDIs and 1 FGD	Kavre district, Nepal		NA	Individuals with hypertension	• Hypertension awareness and treatment. • Health system-related barriers and facilitators of hypertension care utilization.	Research article
32	Tang et al. (63)	Secondary analysis of May Measurement Month	Total = 52,180; Nepal = 14,795	USA, India, and Nepal	Age: 18 years or older	All	Healthy	• Misclassification rates of 1st, 2nd, and contrasting 1st with second (given that 1st measures ≥130/80) by taking at least two measurements	Research article
33	Timilsina (64)	Mixed method study	212	Kathmandu and Kailali district	16 years above	All	Tuberculosis patients	• Prevalence of DM among TB patients	Research article
34	Sharma et al. (65)	Cross-sectional	320	Morang district	15 years and above Male = 214, Female = 96	NA	Clinical	• Prevalence of diabetes • Predictors of the risk factors of Diabetes among the Tuberculosis Patients	Research article
35	Yadav et al. (66)	Interventional time-series of cases	258	Dharan	Male = 123, Female = 135	NA	General OPD patients	• Prevalence of hypertension	Research article
36	Hassan et al. (67)	Secondary analysis of NDHS 2016	3,334	Nepal	>18 years	NA	Hypertensive patients	• Proportion of hypertension awareness among the hypertensive	Research article
37	Kadaria and Aro (68)	Survey (clinic based)	270	Two clinics of Lalitpur and Kaski districts	30–70 years	NA	type 2 diabetes patients	• Prevalence of physical activity; 52% were moderately active and 28% highly active.	Research article
38	Karmacharya et al. (69)	Survey	1,073	Dhulikhel	≥18 years	NA	healthy and clinical	• Proportion of hypertension awareness among the hypertensive • Proportion of taking medication among the hypertensive • Prevalence of Control of HTN	Research article
39	Khanal et al. (70)	RCT	125	Birendranagar municipality of Surkhet district	≥30 years	NA	Hypertnsive patients	• Proportion of taking medication among the hypertensives • Prevalence of Control of HTN	Research article
40	Khanal et al. (71)	Survey	1,159	Birendranagar Municipality of Surkhet district	≥30 years	NA	Clinical and healthy	• Prevalence of HTN • Proportion of hypertension awareness among the hypertensives • Proportion of taking medication among the hypertensives • Proportion of controlled BP among the hypertensives • Prevalence of DM	Report
41	Kibria et al. (72)	Secondary analysis of NDHS 2016	13,519	Nepal	18 years or older	NA	Clinical and healthy	• Prevalence of HTN (as per JNC and ACC/AHA criteria) • Proportion of hypertension awareness among the hypertensives • Proportion of taking medication among the hypertensives • Proportion of controlled BP among the hypertensives	Research article
42	Koirala et al. (73)	Screening at community based setting	140	Community of Dharan	≥18 years	Aryans and Mangolians	Hypertnsive patients	• Proportion of controlled BP among the hypertensives	Research article
43	Koirala et al. (74)	Survey	188	Tsarang village, of Mustang district	≥18–80 years	Highlanders	healthy and clinical	• Prevalence of HTN • Prevalence of DM	Research article
44	Kushwaha and Kadel (75)	Camp survey	114	Community hospital of Kathmandu	>14 years	NA	healthy	• Prevalence of DM	Research article
45	Mehata et al. (76)	Nationally representative cross-sectional study	4,200	Mountain, Hill, and Terai	Adults aged 15–69 years	NA		• Prevalence of metabolic syndrome • Determinants of MetS	Research article
46	Mehata et al. (77)	Secondary analysis of NDHS 2016	13,598	Mountain, Hill, and Terai	Adults aged 15–69 years	NA	Healthy	• Proportion of Hypertension; • Proportion of hypertension awareness among the hypertensives • Proportion of hypertension treatment among the hypertensives • Proportion of hypertension control among the hypertensives	Research article
47	Mishra et al. (78)	Secondary analysis of NDHS 2016	14,823	Mountain, Hill, and Terai	15 years and above (6,245 males and 8,612 females)	NA	Healthy	• Examine the socio-economic inequalities in prevalence, awareness, treatment, and control of hypertension	Research article
48	Mizuno et al. (79)	Cross-sectional study	Total, 1,899; Nepal, 700	Bangladesh, Indonesia, Nepal, and Vietnam	Female (54%) male (46%	NA	Healthy	• Association between urinary heavy metal concentrations and blood pressure among residents of four Asian countries (Bangladesh, Indonesia, Nepal, and Vietnam)	Research article
49	Bista et al. (80)	Secondary analysis of NDHS data 2016	6,396	Mountain, Hill, and Terai	Women of age 15–49 years	Advantage (31.3%) group, Dalit (12.6%), Janjati (36.6%), Other (19.5%)	Healthy	• Prevalence of non-communicable diseases risk factors among reproductive aged women of Nepal • Determinants of non-communicable diseases risk factors	Research article
50	Neupane et al. (81)	Cross-sectional study	123 Female community health volunteers (FCHVs)	Lekhnath municipality, Nepal	20 years and above	Dalit (4.4%), Disadvantaged Janjati (5.3%), Relatively advantaged janajati (9.7%), Upper caste (80.5%)	Healthy	• Knowledge of diagnosis, risk factors, and complications • Attitude toward hypertension and its risk factors • Attitude toward community behavior related to hypertension • Attitude toward future involvement in hypertension management	Research article
51	Neupane et al. (82)	Community-based, open-label, two-group, cluster-randomized controlled trial	1,638 participants (939 assigned to intervention; 699 assigned to control)	Nepal	Adults 25–65 years	NA	Healthy	• Mean systolic blood pressure at 1 year	Research article
52	Neupane et al. (83)	Cross-sectional study	2,815 households	Semi urban area of Lekhnath Municipality,Nepal	Adult population (≥18 years) Female (63%), Male (37%)	NA	Healthy	• Calculate prevalence, awareness, treatment and control level of hypertension.	Research article
53	Niraula et al. (84)	Hospital based cross-sectional study	204 diagnosed patients (102 males and 102 females) with T2DM and 102 healthy controls were enrolled in the study	BPKIHs, Dharan, Nepal			Newly diagnosed and follow-up cases of T2DM v	• Reveal the adenosine deaminase activity in type 2 diabetes mellitus	Research article

**Table 3:** Key findings, strengths, and limitations of included studies.

**SN**	**References**	**Key findings**	**Strengths and limitations**
1	Acharya et al. (33)	• About 31.3% (3592/11481) participants had hypertension. • Among the hypertensive persons, 40.2% (1,444/3,592) were aware of their hypertension status. • Among these who were aware, 79.4% (1,146/1,444) were taking antihypertensive medicine. • However, the overall proportion of hypertensive patients taking medicine was 32.0% (1,146/3,592). • The BP was controlled among 46% (527/1444) of participants who were under medication.	• At least three measures were taken, from which the last two were recorded • Representativeness of the sampling is not mentioned • Geography, caste/ethnicity and age distribution of the participants are not described
2	Adhikari (34)	• Hypertension proportion (Camp) is 70% (1,301/1,857; Pre-HTN, HTN1 and HTN2) • Proportion of hypertension among in-patients is 13.7%	• The report is service coverage based rather than outcome based.
3	Agho et al. (35)	• Prevalence of prehypertension and hypertension was 26.9 and 17.2% respectively • Prehypertension was present in 30.4% (95%CI: 28.7, 32.2) of males and 24.3% (95% CI: 23.1, 25.6) of females, while hypertension was present in 20.4%, (95% CI 18.9, 22.0) of males and 14.8%.	• Nationally representative sample • Lack of temporal relationship with the risk factors and outcome • Limited risk factors considered for study • Post-earthquake situation which might interfere in psychosocial status interfering pre/HTN
4	Bhattarai et al. (36)	• CVDs contributed to 26.9% of total deaths and 12.8% of total DALYs • Ischemic heart disease and stroke were the predominant CVDs, contributing 16 4% (UI, 18.2–14.6) and 7.5% (UI 8.6–6.7) to total deaths and 7.5% (UI, 8.7–6.3) and 3.5% (UI, 4.0–3.0) of total DALYs, respectively.	• This is the first study to report on trends and distribution of the CVD burden at a national level in Nepal.
5	Bist et al. (37)	• The prevalence of raised blood pressure was 24% • The prevalence of raised blood sugar was 5.8% • The prevalence of raised cholesterol was 11% • The proportion of overweight was 24%	• Nationally representative sample
6	Brewis et al. (38)	• The proportion of raised BP is 25.4% among male and 19.3% in female	• Nationally representative sample • The relationship between de/hydration and BP and the direction of effect was measured
7	Aryal et al. (39)	• Proportion of hypertension (including under treatment) is 46.1 and 40.9% in urban and rural areas of Mustang, respectively; and 54.5 and 29.1% in urabn and rural areas of Humla • 30.9% of participants are prediabetic • 6.9% were diabetic • Prevalence of pre-diabetes was significantly higher in rural settings compared to urban settings (*p* < 0.01)	• Selection bias on sampling the survey used non-fasting blood samples for determination of a lipid profile which might interfere with the TG level.
8	Das Gupta et al. (40)	• Prevalence of hypertension was 21% (JNC7) and 44% (2017 ACC/AHA) • Prevalence of hypertension awareness was 37.1 and 43.9% in male and female, respectively. • Prevalence of antihypertensive medication was 47.5 and 50.1% in male and females, respectively • Prevalence of control of hypertension among the hypertensive was 53.5 and 49.2% among male and female, respectively.	• Blood pressure was measured three times in a single day for the study whereas JNC7 guideline recommends longitudinal measurement • Possibly misclassification bias • Robust association between the outcome variables and caste could not be obtained due to missing data
9	Das Gupta et al. (41)	• Overall prevalence of hypertension was 21.1%	• Three blood pressure measurements were recorded; all were done in a single visit within a 5-min interval
10	Datta and Humagain (42)	• Overall prevalence of prehypertensive and hypertensive women were 24.30% and 10.86 whose husband did not consume alcohol•4.5% point gap in hypertension prevalence between wives of alcohol-consuming husbands and those of husbands not consuming alcohol • Likelihood of being hypertensive of Nepalese women was 12.8%	• Husband's alcohol consumption, as a factor of wives' hypertension status.
11	Dhungana et al. (43)	• The most prevalent comorbidity of hypertension and diabetes was 5.7% followed by HTN and COPD (4.8%), and HTN and CKD (4%)	• Secondary analysis of the data from the NCD survey 2018 • Chronic disease multimorbidity determinants and patterns study
12	Dhungana et al. (44)	• Prevalence of hypertension was 34.6% and diabetes 10.5% • 23.2% were not taking any antihypertensive medications among the aware hypertensive patients • 47.2% had controlled blood pressure level among the hypertensive medicine users • Among the Diabetics, only 59.3% were taking medication	• Cross-sectional study • Recall bias while recording dietary and medication history and assessing seven days physical activities
13	Ene-Iordache et al. (45)	• Prevalence of hypertension was 23% in the general population and 38% among high risk cohorts (Framingham risk score) • Prevalence of awareness being onset of disease is 59 and 71% for HTN and Diabetes (74 vs. 56% for HTN and 80 vs. 69% for Diabetes among high risk cohorts and general population) • Self-reported of onset of having disease is 11 and 4%, respectively among high risk cohorts and general population	• Individuals were screened based on convenience sampling, section bias on recruiting volunteers who will provide the testing
14	Ghimire et al. (46)	• Proportion of Hypertension was 57.2% • Proportion of Diabetes was 15%	• Nationally representative survey data • Age group of only 60–69 years, though >30% population is over 70 years, so generalizability is limited.
15	Ghimire et al. (47)	• Prevalence of raised blood pressure was 31.4%	• Self-reporting of disease status • Non-response rate-21.84%
16	Gyawali et al. (48)	• Mean cost case treatment was ranged from 484.8 to 445.9 USD per annum and per visit 5.1–16.2 USD • Prevalence of DM Type 2 ranges from 4.5% to as high as 19% in urban Nepal and rural prevalence ranging from 0.3 to 2.5%	• Costing study of DM • Limited to the selected database source • Urban-focused
17	Paudel et al. (49)	• Almost one-fourth (29.49%) of the adult population in the community suffered from hypertension. • Less than half (43.2%) of the hypertensive patients were aware of their conditions • 94.9% were taking antihypertensive medication and 68.4% had their blood pressure controlled	• This study is one of the few studies of Kaski district, Nepal to assess the awareness, treatment and control status of hypertensive patients. • This study limits its scope as the causal inferences could not be drawn. • This study is based on one district of Nepal and is not representative of the whole country due to the high ethnic, dietary, cultural, and geographical variation in the country
18	Peoples et al. (50)	**Quantitative finding** • Medicine cost was rated “too expensive” by 52 and 63% of rural and urban participants, respectively. • Perceived poor bedside manner was tied to negative perceptions of PHC quality, and vice versa. • Lack of resources and excessive barriers to care were mentioned by every interviewee. **Qualitative finding** • PHC use was high and satisfaction was low. • Most of the people were found unaware and didn't have any idea how to manage the disease when they were interviewed	• The study is claimed to be the first to examine perception and use of PHC services for Cardiometabolic diseases (CMDs) in Nepal. • The use of only two districts of Nepal as study sites, the use of cluster convenience sampling, and the limited sample size.
19	Rana et al. (51)	• Women were having lower prevalence of hypertension compared with men for both measured (16.0%, 95% CI: 14.8, 17.3 vs. 22.8%, 95% CI: 21.2, 24.5) and medical hypertension (21.7%, 95% CI: 20.4, 23.0 vs. 29.1%, 95% CI: 27.4, 30.8) and the differences were significant statistically in both measurements (*p* < 0.001). • People living in urban areas were having higher prevalence of hypertension compared with people living in rural areas for both measured (19.5%, 95% CI: 18.7, 20.4 vs. 17.9%; 95% CI: 16.9, 19.0) and medical (26.2%, 95% CI: 25.2, 27.1 vs. 22.7%; 95% CI: 21.6, 23.8) hypertension and the differences were significant statistically (*p* < 0.001) only for medical hypertension. • There was an overall 21% increase in the prevalence of hypertension, with the highest increase in the male population (23%)	• Assessed the association between SES and hypertension according to standard hypertension JNC7 guideline and a new guideline recommended by the ACC/AHA 2017. • The study could not assess the causality of the associations between Socio Economic Status and hypertension due to the cross-sectional nature of the data
20	Rai et al. (52)	• Hypertension was the common systemic disease associated in 40.8% of the cases, followed by diabetes in 32.5% and combined diabetes and hypertension in 20.2%. • Wealthy urban population in Nepal had higher prevalence, awareness, treatment and control than the poorer and poorest population. • The odds of being hypertensive was higher in men compared to women 1.96 (1.59–2.44) for Nepal)	• Recorded data was analyzed for HTN and DM, of the eye patients visiting a tertiary eye care center • Ocular co-morbidities have not been included, so a certain proportion might have been missed
21	Rauniyar et al. (31)	• Prevalence of hypertension in Nepal was 19.6%. • Less than one-third (20.2%) of the hypertensive population received treatment and 10.4% among them had their blood pressure controlled. • The odds of being hypertensive was higher in men compared to women1.96 (1.59–2.44). • Prevalence of hypertension was 7.1 (2.9–11.4) percentage points higher in affluent populations compared to the disadvantaged ones in Nepal.	• Provides detailed information on existing inequalities in prevalence and management of hypertension • The study cannot be generalized to population aged 50 years and above
22	Sainju et al. (53)	• Pre-hypertension and hypertension were seen in 11.02 and 30.17% of the study population, respectively • Almost three-fifths of the obese participants were hypertensive	• Sample is not nationally representative • Single episode of measurement of blood pressure (three readings) was taken, which may not be sufficient to diagnose hypertension in the population. • There could be an error due to observer variation in hearing the Koratkoff sound in crowed places
23	Saito et al. (54)	• Prevalence of hypertension (36.7%) • Prevalence of diabetes (14.4%)	• Self-reported assessment of illness may be biased • Face-to-face interview (only by asking) may not suffice to assess all the NCD risk factors • Data was collected in winter season, which might have affected the prevalence
24	Paudel et al. (55)	• 9% had diabetes with the prevalence higher among males (12.7%) than females (6.9%) • Overweight and obesity, Waist Circumference >102 cm (males) or >88 cm (females), a triglyceride level ≥150 mg/dL and total 14 cholesterol ≥190 mg/dL were associated with Type 2 Diabetes Mellitus	• Measurement and modeling of multiple behavioral, socio-economic and biological risk factors assessed • STEP survey data of 2013 re-analyzed for 40–69 yrs age group
25	Gyawali et al. (56)	• Prevalence of type 2 diabetes 11.7% (95% CI: 10.4–13.1) • Prevalence of prediabetes 13.0% (95% CI: 11.8–14.5) • Nearly two-fifths (35%) unaware of their disease • Nearly 94% of those aware were receiving some kind of treatment such as insulin or oral anti-diabetic medications and counseling • Control rate was less than one quarter of those who were receiving treatment (21%)	• One of the few studies on the awareness, treatment and control of diabetes in Nepal through validated STEPS questionnaire and fasting blood glucose measurements according to the WHO recommendations • The use of self-reported physical activity measures that are subjected to recall bias and over-reporting could have increased the possibility of exposure misclassification
26	Shrestha et al. (57)	• The prevalence of T2DM, pre diabetes, and impaired glucose tolerance in Nepal was estimated to be 10, 19.4, and 11%, respectively. • Normal waist circumference, normal blood pressure and no history of T2DM in a family has 64.1, 62.1, and 67.3%, respectively	• Heterogeneity in the studies due to variation in the T2DM diagnostic criteria and different demographics of the population
27	Shrestha et al. (58)	• Prevalence of prediabetes and diabetes was 9.2% (95% CI 6.6–12.6%) and 8.5% (95% CI 6.9–10.4%), respectively. • 52.7% (95% CI 41.7–63.4%) were aware of their diabetes status. 45.3% (95%CI 31.6–59.8%) were taking antidiabetic medications.	• High heterogeneity between the reported diabetes prevalence across the included studies
		• Nearly one-third of those under antidiabetic treatment (36.7%; 95% CI 21.3–53.3%) had their blood glucose under control	
28	Shrestha et al. (59)	• Prevalence of hypertension and pre-pre hypertension was 40.67 and 36.77%, respectively • Age AOR for being hypertensive for males compared with females was 0.86 (95% CI 0.72–1.02) • Sex AOR for being hypertensive was 1.61 (95% CI 1.35–1.91) for the age group 55–69 compared with age group 40–54 years. • Participants with WC measures greater than the cut-off value were twice as likely to be hypertensive (2.02; 95%CI 1.66–2.45) than people with normal WC	• Waist to height ratio and waist circumference were also included for picking up obesity with higher cardiovascular risk despite normal body mass index • Underlying causes and co-morbidities are not included
29	Silvanus et al. (60)	• Prevalence of known diabetes (50/306) was an estimated 16.34% (95% CI: 12.62% to 20.90%) • 46.09% were classified as high risk, 44.53% as moderate risk and 9.38% as low risk for developing diabetes • The tabulated sensitivity (true positive rate) of the IDRS cut-off score ≥60 (high risk classification) was found to be 84.21% with a specificity (true negative rate) of 55.24% • The false positive rate and false negative rate was 44.76 and 15.79%, respectively. • The positive predictive value was 20.0% and negative predictive value was 96.34%	• Community-based study design to screen for undiagnosed diabetes, the step wise approach including the non-invasive tool and estimation of RCBG and the use of both FPG and the 2 h PG following a 75 g OGTT to identify diabetes and prediabetes • A community-based screening program can attract persons who have the health condition, those with a propensity to seek health care or who are more interested in their health which can introduce a selection bias
30	Silvanus et al. (61)	• Prevalence of undiagnosed diabetes was 4.32% (95% CI 1.75–8.70%) and that of prediabetes was 7.14% (95% CI 3.89–12.58%) • The overall prevalence of persons with “raised blood glucose” was 11.73% (95% CI 5.64–21.28%) • All of the persons with prediabetes (*n* = 12) had IGT	• Recognizing the use of glucometer and capillary sampling in low- and middle-income countries • Two glucometers were used during the screening camp, within glucometer variation was not studied
31	Tan et al. (62)	• Most individuals with hypertension could link hypertension to its causes, symptoms and complications • Some individuals with hypertension occasionally stopped medication due to forgetfulness, negligence, laziness, and affordability issues	• First qualitative study in Nepal involving a range of stakeholders to gather multidimensional insights into hypertension management • Qualitative design and small sample size limit the generalizability of the study findings
32	Tang et al. (63)	• The range of 8.2–12.1% and 4.3–9.1% missed and overidentified hypertensive, respectively found when only 1st measurement was taken. • The range of 4.9–6.4% and 2.0–4.4% missed and overidentified hypertensive, respectively, found when only 2nd measurement was taken but for this, all the participants needed to go through screening. • The range of 3.8–8.1% and 2.0–3.9% missed and overidentified hypertensive, respectively, found when 2nd measurement was taken only for those having BP ≥ 130/80 during 1st screening. The range (%) of the participants needed to screen in the conditional screening (BP ≥ 130/80) for 2nd time is only 33.8–59.8% • Hence, resource cost is reduced by 40.2–66.2% when conditional sequential screening is carried out	• Comparison of 1st, 2nd, and conditional 3rd screening was carried out so as to assess the difference in resources used • Survey data of USA is taken from 1999–2016 whereas findings from opportunistic screenings of May Measurement Month of 2017–18 for India and Nepal were used
33	Timilsina (64)	• Prevalence of DM among TB patients was 18.84%.	• Sample was taken purposively
34	Sharma et al. (65)	• The prevalence of diabetes, pre-diabetic and glucose intolerance among tuberculosis patient was 11.9, 17.2, and 17.8%, respectively. • Current alcohol consumer as the significant predictor of diabetes among the tuberculosis patient	• The Fasting Blood Sugar and 2-h Post-Prandial Blood Sugar were assessed by the glucose oxidase method • Facility-based DOTS center sample was taken
35	Yadav et al. (66)	• 56% were diagnosed as hypertensive; • 29% were pre-hypertensives; • 16.3% had 1st stage hypertension and 11% had 2nd stage hypertension	• The study sample was obtained from the tertiary level teaching hospital • Less generalizability at population
36	Hassan et al. (67)	• Among the total hypertensive participants, identified only in NDHS 2016 survey but not by professionals earlier, prevalence of diagnosed hypertension was -Total, 49.6%; -Province 1, 53%; Madhesh Province, 53.1%; Bagmati Province, 52.7%; Gandaki Province, 46.9%; Lumbini Province, 45.4%; Karnali Province, 39.8%; SudurPaschim Province, 41.9% • Mountain, 47.1%; Hill, 48.3%; Terai, 51.4% • Prevalence of undiagnosed hypertension was 50.4% • Proportion of hypertension awareness among the hypertensives was 49.6%. • Undiagnosed hypertension was disproportionately higher among lower socioeconomic status groups (Concentration Index, *C* = −0.18, *p* < 0.001).	• Nationally representative, cross-sectional data to determine the prevalence • Other behavioral and lifestyle factors potentially relevant to undiagnosed hypertension, for example, physical activity, dietary patterns and family history of hypertension, that were not explored in this study.
37	Kadaria and Aro (68)	• 52% were moderately active • 28% were highly active	• Facilitators and barriers physical activity were assessed • Self reporting and its recall bias on measure of physical activity
38	Karmacharya et al. (69)	• Proportion of hypertension awareness among the hypertensives was 44.7% • Prevalence of taking antihypertensive treatment was only 76.1% (among the known hypertensives and 33.2% among the total) • Prevalence of control of hypertension was 35.3% among the known hypertensive and 11.7% of the total	• Spectrum of awareness, treatment and control of hypertension • BP was taken in home setting and single day measurement cause false readings and affect the study • The targeted study was conducted at Dhulikhel which has teaching hopsital that could impact on level of awareness and control of HTN among the participants.
39	Khanal et al. (70)	• Proportion of participants controlling Systolic BP increased to 58.3% from 3.3% compared to only to 40% among the intervention vs. control group • Percentage of the controlled Diastolic BP increased by 30% after the intervention compared to only 20% on usual care (control)	• The study was study was conducted in one municipality and high number of female respondents thus limited generalization. • The blood pressure measured twice at 3-min interval in a single visit
40	Khanal et al. (71)	• Prevalence of hypertension was 38.9%2 • 53.4% were aware about their HTN status	• The study was conducted in one municipality and high number of female respondents thus limited generalization
		• 29% on treatment among the hypertensive, and • 8.2% had controlled blood pressure among the treated • Self-reported prevalence of Diabetes was 6.9%	• The blood pressure measured twice at 3-min interval in a single visit • Presence of diabetes was determined as reported by participants without blood sugar measurement
41	Kibria et al. (72)	• HTN prevalence, 44.2% (as per 2017 ACC/AHA) but only 21.2% (as per JNC 7 guideline) • HTN awareness proportion, 40.4% (as per 2017 ACC/AHA) but only 23.6% (as per JNC 7 guideline) • 20.4 vs. 9.8% (as per JNC vs. 2017 ACC/AHA category) of those who would have been considered hypertensive were taking antihypertensive medications • Among the hypertensives, about 9.7 and 7.2% had a controlled blood pressure level, respectively (as per JNC vs. 2017 ACC/AHA category)	• The survey data was nationally representative • Blood pressure of the participants was measured 3 times in a single day while both guidelines recommend the longitudinal measurement • Comparison and effectiveness of two methods of BP measurement and classification
42	Koirala et al. (73)	• Proportion of controlled BP among the hypertensives was 75%	• Sample size is low and generalizability is limited.
43	Koirala et al. (74)	• 20.7% of participants were hypertensive Proportion of Intermediate Hyperglycemia was 31.6 and 4.6% was of DM based on Hba1C measure	• Sample size is low and generalizability is limited due to single village taken for sampling • Fasting blood sugar was not taken for confirming DM diagnosis
44	Kushwaha and Kadel (75)	• Prevalence of diabetes mellitus was found as 4.38%.	• Glucometer with glucose sticks was used to measure the random blood sugar level which was not recommended in respect to fasting blood glucose with biochemistry method
45	Mehata et al. (76)	• The overall prevalence of MetS is 15 and 16% according to Adult Treatment Panel III (ATP III) and International Diabetes Federation (IDF) criteria, respectively • Triad of low HDL-C, abdominal obesity and high BP was the most prevalent (8.18%), followed by abdominal obesity, low HDL-C cholesterol and high triglycerides (8%)	• Provides the first nationally representative estimates on prevalence, disaggregated by sub-groups, and factors attributed to metabolic Syndrome among adult population of Nepal
46	Mehata et al. (77)	• Prevalence of hypertension was 18% (95% CI 16.7–19.2) • Among the total hypertensive individuals, only 38% were aware of their hypertensive status • 18% were taking antihypertensive medication • Half of the hypertensive participants on treatment (52%) had their blood pressure under control.	• Based on a large national sample consisting of both urban and rural populations in Nepal • Dietary habits, alcohol intake or physical activity as the major determinants of hypertension status could not be explored
47	Mishra et al. (78)	• Prevalence of hypertension was 19.5% (95% CI: 18.3–20.7) • Of total hypertensives, the prevalence of hypertension awareness, treatment and control was 40.0% (95% CI: 37.5–42.6), 20.2% (95% CI: 18.0–22.2) and 10.5% (95% CI: 8.8–12.2), respectively	• First nationwide study to examine socio-economic disparities in hypertension burden and cascade of services
48	Mizuno et al. (79)	• Hypertension was 23% • The urinary lead concentrations were positively associated with both systolic and diastolic blood pressure. • Urinary selenium concentrations were negatively associated with both systolic and diastolic blood pressure.	• Wide variation of data (17 communities with various characteristics across four Asian countries) • Association of heavy metals (Pb and Se) were associated with hypertension
49	Bista et al. (80)	• 22.2% were overweight and obese • 11.5% of the participants were hypertensive. • Around 6% of participants had co-occurrence of two NCDs risk factors.	• Adjusted prevalence ratio (APR) was calculated from multiple poisson regression method • Secondary data analyzed for reproductive aged women
50	Neupane et al. (81)	• Low, medium, and high levels of knowledge about hypertension were 43, 24, and 31%, respectively • No significant differences were observed in the knowledge and attitudes related to hypertension in relation to demographic characteristics of FCHV. • A majority of FCHV agreed that smoking (69.8%), alcohol (77.8%), low physical activity (42.4%), high salt intake (65.4%), high fat intake (78.7%), and genetics (53.9%) are major risk factors for hypertension.	• The study was conducted only among FCHV based in 1 municipality in Nepal
51	Neupane et al. (82)	• HTN was 29.6%, *M* = 55.4%; *F* = 24.1 • Pre-HTN was 20.6% • The mean systolic blood pressure at 1 year was significantly lower in the intervention group than in the control group for all cohorts: the difference was −2·28 mm Hg (95% CI −3·77 to −0·79, *p* = 0·003) for participants who were normotensive, −3·08 mm Hg (−5·58 to −0·59, *p* = 0·015) for participants who were prehypertensive, and −4·90 mm Hg (−7·78 to −2·00, *p* = 0·001) for participants who were hypertensive	• First cluster-randomized controlled trial to report systolic blood pressure among normotensive, prehypertensive, and hypertensive populations through an existing network of community health workers
52	Neupane et al. (83)	• The age and sex adjusted prevalence of hypertension was 28% • Among hypertensive participants, 46% were aware of their preexisting hypertension, 31% were on hypertensive medication, and 15% met BP control targets • Increasing age (1.07, 95% confidence interval: 1.06; 1.08), higher body mass index (OR: 1.09, 95% CI: 1.06; 1.12), men (OR: 1.63, 95% CI: 1.25; 2.14), harmful alcohol intake (Or: 2.46; 95% CI: 1.73; 3.51), family history of hypertension (OR: 1.42; 95% CI: 1.14; 1.76), and diabetes (OR: 2.08, 95% CI: 1.30; 3.33) were independently associated with hypertension	• High response rate, adequate representation of both sexes, utilizing average of two BP measurements preceded by a first disregarded measurement and detailed information on the history of hypertension, and pharmacological treatments.
53	Niraula et al. (84)	• Serum ADA levels (U/L) was significantly raised in Uncontrolled Diabetic patients (49.24 ± 16.89) compared to controlled population (35.74 ± 16.78) and healthy controls (10.55 ± 2.20), *p-*value < 0.001 • A significant positive correlation was obtained between Serum ADA and HbA1c, Fasting Plasma Glucose and Post-prandial Glucose respectively	• Serum Adenosine deaminase (ADA) level can also be used as a biomarker in predicting glycemic control in diabetic patients • ADA level also indicates the presence of other diseases • Hospital based comparative cross-sectional study • Convenient sampling

## Results

After excluding duplicates, irrelevant studies, and further eligibility assessment, 53 articles were included in the scoping review.

Of the 53 included studies, 11 reported both DM and HTN prevalence. Participants aged 50 years and older from Bagmati Province and Terai had a higher prevalence of DM. Similarly, people aged 60–69 years from Karnali Province and urban hills had a higher prevalence of HTN. A higher DM prevalence was reported among the clinical participants. For HTN, it was higher among both healthy and clinical participants. Similarly, studies/screenings carried out in ambulatory settings had a higher prevalence for DM, but for HTN, it was reported to be higher in the camps. Males had a higher prevalence among participants with DM and HTN, and co-morbidity for both diseases ranged from 5.7 to 20.2%. Interestingly, pre-HTN and pre-DM were reported among about one-third of the participants, and importantly, for both DM and HTN, a minimum of one-fourth were unaware, and among those on medication, a maximum of only about half had their diseases under control (Table 4; Figure 2).

**Table 4:** Disease-wise summary prevalence/proportion (range^*^) of included studies.

**SN**	**Study characteristics and demographics**	**DM**		**HTN**	
		**Min-Max**	**Ref**	**Min-Max**	**Ref**
1	**Samples**				
	Primary studies	114–9,177	[55, 76]	123–11,486	[33, 82]
	Secondary analysis	526–21,066	[45, 46]	526–21,066	[45, 46]
2	Total (M; F)	114–14,857 (58–6,245; 56–8,612)			
3	**Study types**				
	Total (*n* = 53)^$^	21		44	
	Primary study (*n* = 31)	15		22	
	Secondary analysis (*n* = 21)	4		20	
	Review studies (*n* = 3)	1		2	
	Interventional studies (*n* = 3)	0		3	
	Qualitative (*n* = 1)	0		1	
	Mixed-methods studies (*n* = 2)	2		1	
4	**Sex-wise distribution**				
	Male	12.7	[56]	22.8–55.4	[51, 83]
	Female	6.9	[56]	10.9–43.9	[40, 42]
5	**Age**				
	General (all)	4.4–18.8	[65, 75]	17.2–70.0	[34, 35]
	15–49 yrs	-		10.9–19.6	[42, 53]
	15–69 yrs	9.0	[56]	8.2–31.4	[47, 77]
	18 yrs+	4.6–11.7	[57, 75]	5.7–54.5	[39, 43]
	18–70 yrs	-		34.6	[44]
	40–69 yrs	-		40.6	[60]
	50 yrs+	16.3	[61]	-	
	60–69 yrs	15.0	[46]	57.2	[46]
6	**Geographic distribution**				
	National (more than 1 province)	5.7–15.0	[43, 46]	5.7–70.0	[34, 43]
	Province 1	11.9	[66]	56.0	[67]
	Madhesh	-		53.1^#^	[68]
	Bagmati	4.4–32.5	[52, 76]	30.2–40.8	[52, 54]
	Gandaki	4.6–11.7	[57, 75]	20.7–46.1	[39, 75]
	Lumbini	-		45.4^#^	[68]
	Karnali	6.9		29.1–54.5	[39]
	Sudur-Paschim	18.8	[39]	41.9^#^	[68]
	Urban	-	[65]	46.1–54.5	[39]
	Rural	-		29.1–40.9	[39]
7	**Ecological belts**				
	Mountain	4.6–6.9	[39, 75]	20.7–54.5	[39, 75]
	Hill	4.4–16.3	[61, 76]	28.0–56.0	[67, 84]
	Terai	11.9–18.8	[65, 66]	51.4^#^	[68]
8	**Participants**				
	Healthy	4.4–16.3	[61, 76]	18.0–36.7	[55, 78]
	Clinical	18.8	[65]	13.7–56.0	[34, 67]
	Both	5.7–15.0	[43, 46]	5.7–70.0	[34, 43]
9	**Types of study/screening settings**				
	Survey/evidence synthesis	5.7–14.4	[43, 55]	5.7–57.2	[43, 46]
	Ambulatory	11.9–18.8	[65, 66]	40.6–40.8	[52, 60]
	Opportunistic	-		31.3	[33]
	Camp	4.4	[76]	70.0	[34]
10	**Disease awareness and control**				
	Awareness	35.0–80.0	[45, 57]	23.6–74	[73, 45]
	On medication (among aware)	45.3–94.0	[57, 59]	9.8–94.9	[49, 73]
	BP/Sugar control (by medicine)	21.0–36.7	[57, 59]	8.2–52.0	[72, 78]
	BP/Sugar control (overall)	-		9.7–68.4	[49, 73]
	On medication (overall)	59.3	[44]	18.0–66.8	[44, 78]
11	**Pre-and undiagnosed proportions**				
	Pre-diabetes/pre-HTN	9.2–31.6	[59, 75]	11.2–36.8	[54, 60]
	Undiagnosed Pre-DM/HTN	7.1	[62]		[68]
	Undiagnosed DM/HTN	4.3	[62]	50.4^#^	
12	**Co-morbid status**				
	HTN and DM	5.7–20.2	[43, 52]		
	HTN and COPD	4.8	[43]		
	HTN and CKD	4.0	[43]		

* In some variables, only single value could be extracted.

$ Some studies were common for DM and HTN.

# Among the total hypertensive participants, identified only in NDHS 2016 survey but not by health professionals earlier, as diagnosed and undiagnosed.

**Figure 2:**
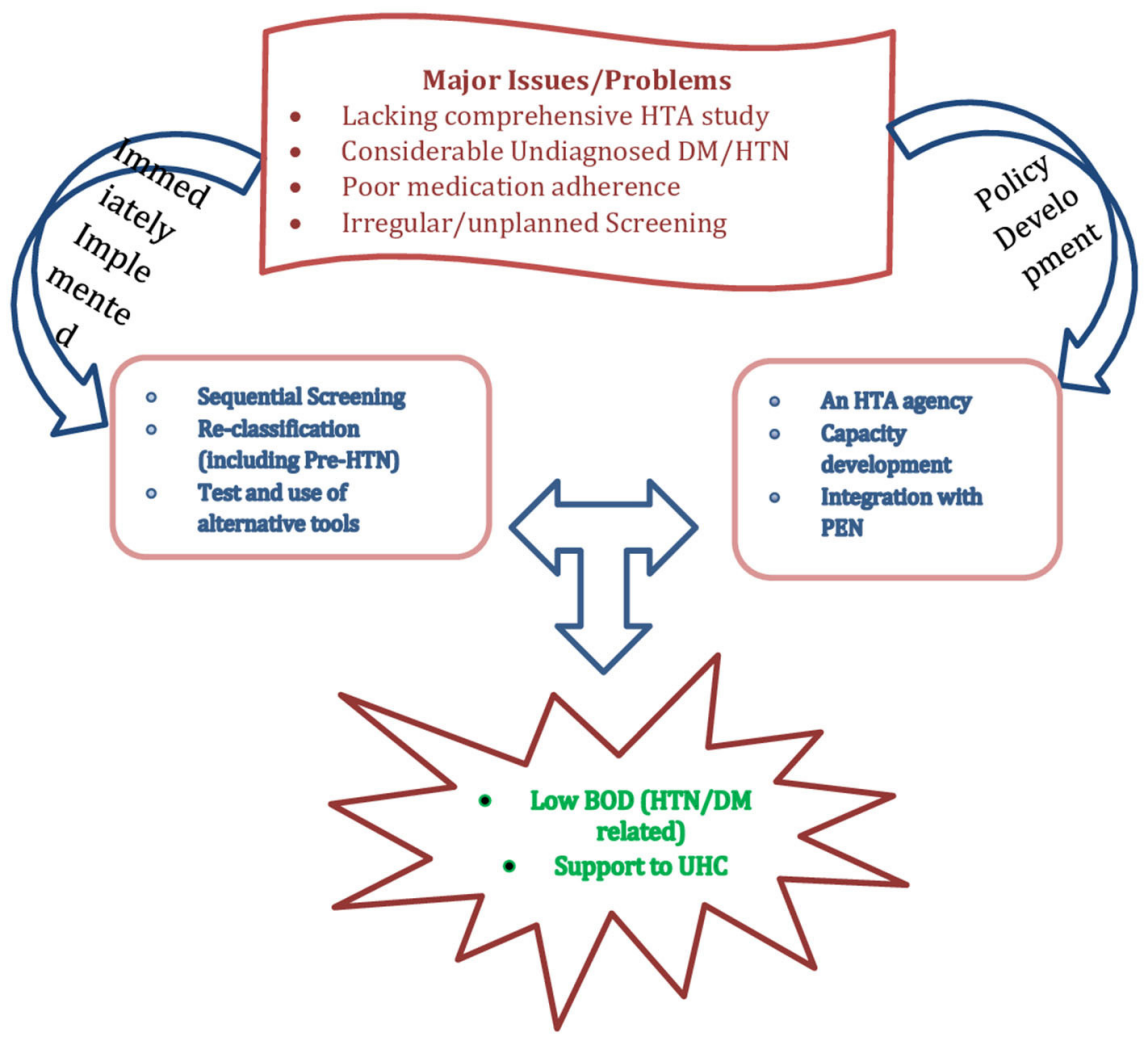
Summary of findings.

### Validation and alternative use of the technology

The Indian Diabetes Risk Score (IDRS) is a simple tool that includes four statements to screen for diabetes and, with a cut-off score of 60, among the Nepalese population, revealed a sensitivity and a specificity of 84.2 and 55.2%, respectively, and hence could be a suitable alternative for diabetes and prediabetes screening (60). A hospital-based study revealed that measurement of serum Adenosine Deaminase (ADA), a biomarker, can be an alternative tool for glycaemic control and monitoring. It showed a significant correlation with HbA1c, fasting blood sugar (FBS) and post-prandial glucose (84). Similarly, a simple technological change in classification in the national guideline, changing the category of (130–140)/(80–90) mm of Hg as pre-HTN as defined by the Seventh Report of the Joint National Committee on Prevention, Detection, Evaluation, and Treatment of High Blood Pressure (JNC 7) to HTN as newly defined by the American Heart Association/American College of Cardiology (AHA/ACC), can reduce cardiovascular and related health problems by early detection and thereby save more lives and money (40). Another application of sequential screening, BP ≥130/80 mm of Hg, if assessed a second time (without assessing all for a second time), would give almost the same result but reduce the cost (time, human resources) by 40–60% during the camp and other opportunistic screenings (63). Moreover, the relationship of heavy metals with BP indicates that we need to address the problems of NCD prevention and control from a different perspective. On the other hand, HTN and DM are both webbed with multiple risk factors. For example, the triad of HDL-C, abdominal obesity and HTN was found among nearly 1 in 10 (8.2%) persons (76), and 6% women of reproductive age had co-occurrence of two NCD risk factors. This implies that these problems the rather be addressed with multiple and complex interventions, along with some feasible policy interventions (Figure 2).

### Cost, DALYs, and equity

The prevalence of HTN was found to be more than seven (7.1) times higher among the wealthy than among the poor (31) but undiagnosed HTN was found to be disproportionate (1.6 times higher) among those with lower socioeconomic status (*C* = −0.18, *p* 0.001) (67). According to a qualitative finding, 52% of rural and 63% of urban people considered the medicine used to treat HTN and DM to be expensive (50). A review of costing studies showed that in DM, USD 445.9 (±27,535), 16.2 and 5.1 are needed per annum per patient, per prescription, and per visit per patient, respectively (48), thus, implicating medication non-adherence (Figure 2).

### Awareness, medication adherence, and intervention efficacy

Nine out of every 10 people have moderate to high chances of developing DM (60), which indicates the importance of early interventions. HTN medication was missed due to forgetfulness, negligence, and unaffordability (62), which could be attributed to a lack of or poor HTN knowledge among community members (50) and the female community health volunteers (FCHVs) (81). However, when these cadres were trained and prepared as change agents, the intervention could reduce a mean blood pressure by 2.3–4.9 mm Hg and increase the proportion of BP control and knowledge of the community members (70, 82).

## Discussion

Overall, the prevalence of high blood pressure was found to be as low as 5.7% (43) and as high as 70% (46) nationally, and up to 55% (39) in Karnali province and in urban areas. Similarly, DM was found to be between 5.7 (43) and 15% (46) nationwide, though it was as low as 6.9% (39) and as high as 32.5% (52) in Karnali and Bagmati provinces, respectively. Similarly, there is also a wide variation in DM (45–94%) and HTN (9–95%) awareness (49, 56, 58, 72), disease/disorder controlled by medicine (56, 58, 71, 77) in the population. This variation should be considered when planning and implementing the relevant programs for coverage and efficacy (Figure 2). A combined study of systematic review and expert consultation carried out in eight European countries found that the incorporation of additional social value judgments beyond clinical benefit assessment and economic evaluation could help further explain heterogeneity in coverage recommendations and decision-making (85). Similarly, it would be wise to compare the cost in lieu of the yield and the diagnoses that the screening strategies give. In an ambulatory high-risk approach carried out in the Bhutan PEN evaluation study (19), a 10% of eligibility and around 23% of diagnosis for HTN and a 10% of eligibility and 26% of diagnosis for DM may be contrasted with a 13% of eligibility and 17% of diagnosis for HTN and a 19% of eligibility and 22% of diagnosis in an opportunistic high-risk screening approach carried out in Karnataka, India (20). In these two studies, number of diagnoses was higher in ambulatory high-risk approach but yield was higher in opportunistic high-risk approach.

Producing HTA capacity in a country like Nepal may run up against different problems at various stages, such as policy sensitization, development and implementation, expertise development, and overall management. A similar program carried out by the Health Intervention and Technology Assessment Program (HITAP) of Thailand in India, Colombia, Myanmar, the Philippines, and Vietnam revealed experiences suggesting that it is not only technical capacity, such as analytical techniques for conducting economic evaluation, but also management, coordination, and communication capacity should be strengthened (86). Inequality in HTN was observed to have up to 1.6 times higher prevalence among the poorest and underprivileged (31, 67). A combined study of documentary review and interviews carried out by Ciani et al. in Italy during the early stage of HTA development, argued that not only the central agency should be in place, but also a fully coordinated and harmonized multi-level structure of HTA was imperative (87). Then, the Italian National Healthcare System was one of the most decentralized systems since the devolution reform approved in 2001, and Nepal is more or less, at present, in a similar situation with devolution of power to the local levels with a 3-tier governing system.

Regarding the universal health coverage (UHC) and social health security program (SHSP) that are underway, we are adopting and scaling-up different benefit packages by assessing only a few to many, but not all components. The early results of Thai universal coverage in 2009–10 showed that HTA is helpful for informing coverage decisions for health benefit packages because it enhances the legitimacy of policy decisions by increasing the transparency, inclusiveness, and accountability of the process (88). The National Institute for Health Research HTA programme also suggests that synthesized evidence, rather than depending upon clinical trials, should be taken into account, for decision analysis and that the findings of a trial be linked to systematic reviews and meta-analyses (89).

## Limitations

The included studies' principal limitations were costing comparisons of technologies in light of comparative validity and precision. Furthermore, there was a lack of ethical, ICER, quality of life, and willingness and capacity to pay for various technologies.

## Conclusion

Overall, 53 studies, including some secondary analysis and the reviews, mainly reported the prevalence of DM of 4.4–18.8% and HTN of 17.2–70%. In addition to establishing an HTA national agency, some immediate actions and a systematic HTA of both diseases, covering a representative sample, is highly recommended before warranting.

## Author contributions

CA, KSh, RD, and LA conceptualized the review, developed the search strategy, the data extraction grids, and edited. CA, LA, and BP curated data. CA, KSh, and KSu carried out the formal analysis. CA, BP, LA, and AT extracted the data. CA and KSh administered, supervised, and validated the project. CA, RD, LA, BP, KSu, AT, YA, and KSh wrote the draft. All authors reviewed the final manuscript and agreed for submission.

## Conflict of interest

Author LA was employed by HERD International. The remaining authors declare that the research was conducted in the absence of any commercial or financial relationships that could be construed as a potential conflict of interest.

## Publisher's note

All claims expressed in this article are solely those of the authors and do not necessarily represent those of their affiliated organizations, or those of the publisher, the editors and the reviewers. Any product that may be evaluated in this article, or claim that may be made by its manufacturer, is not guaranteed or endorsed by the publisher.
